# Factors associated with hospitalization in a pediatric population of rural Tanzania: findings from a retrospective cohort study

**DOI:** 10.1186/s13052-024-01622-z

**Published:** 2024-03-18

**Authors:** Vincenzo Mancini, Martina Borellini, Paolo Belardi, Maria Carolina Colucci, Emanuel Yuda Kadinde, Christina Mwibuka, Donald Maziku, Pasquale Parisi, Anteo Di Napoli

**Affiliations:** 1 Chair of Pediatrics, NESMOS department, Faculty of Medicine & Psychology, Sapienza University, Rome, Italy; 2 Doctors with Africa CUAMM, Iringa, Tanzania; 3 Tosamanga Voluntary Agency Hospital, Tosamanga, Tanzania; 4 Epidemiolgy Unit, National Institute for Health Migration and Poverty (INMP), Via di San Gallicano, 25a - 00153 Rome, Italy

**Keywords:** Pediatrics, Emergency services, Hospitalization, Predictive factors, Low-income country, Tanzania

## Abstract

**Background:**

Despite pediatric acute illnesses being leading causes of death and disability among children, acute and critical care services are not universally available in low-middle income countries, such as Tanzania, even if in this country significant progress has been made in child survival, over the last 20 years. In these countries, the hospital emergency departments may represent the only or the main point of access to health-care services. Thus, the hospitalization rates may reflect both the health system organization and the patients’ health status. The purpose of the study is to describe the characteristics of clinical presentations to a pediatric Outpatient Department (OPD) in Tanzania and to identify the predictive factors for hospitalization.

**Methods:**

Retrospective cohort study based on 4,324 accesses in the OPD at Tosamaganga Voluntary Agency Hospital (Tanzania). Data were collected for all 2,810 children (aged 0–13) who accessed the OPD services, within the period 1 January − 30 September 2022. The association between the hospitalization (main outcome) and potential confounding covariates (demographic, socio-contextual and clinical factors) was evaluated using univariate and multivariate logistic regression models.

**Results:**

Five hundred three (11.6%) of OPD accesses were hospitalized and 17 (0.4%) died during hospitalization. A higher (*p* < 0.001) risk of hospitalization was observed for children without health insurance (OR = 3.26), coming from more distant districts (OR = 2.83), not visited by a pediatric trained staff (OR = 3.58), and who accessed for the following conditions: burn/wound (OR = 70.63), cardiovascular (OR = 27.36), constitutional/malnutrition (OR = 62.71), fever (OR = 9.79), gastrointestinal (OR = 8.01), respiratory (OR = 12.86), ingestion/inhalation (OR = 17.00), injury (OR = 6.84).

**Conclusions:**

The higher risk of hospitalization for children without health insurance, and living far from the district capital underline the necessity to promote the implementation of primary care, particularly in small villages, and the establishment of an efficient emergency call and transport system. The observation of lower hospitalization risk for children attended by a pediatric trained staff confirm the necessity of preventing admissions for conditions that could be managed in other health settings, if timely evaluated.

**Supplementary Information:**

The online version contains supplementary material available at 10.1186/s13052-024-01622-z.

## Background

Annually, more than 80 per cent of the 6.4 million deaths in children under 14 years of age occur in low- and middle-income countries (LMICs) [1]. These deaths are predominantly due to acute illnesses (such as sepsis, pneumonia, infections, trauma, etc.) that could be successfully managed with basic intensive care interventions, including fluid resuscitation, ventilatory support and blood product transfusion [2, 3]. Although pediatric acute illnesses are globally the leading causes of death and disability for children outside the neonatal period, acute and critical care services remain inconsistently available in resource-limited settings [2–6]. Therefore, it is essential to gather specific data on the etiology of acute critical illness to facilitate the development of evidence-based intervention plans and proper allocation of the available yet limited resources in LMICs.

Tanzania is a LMIC in sub-Saharan Africa [7]. Over the last 20 years, significant progress has been made in child survival, bringing Tanzania close to the target set by the Millennium Development Goals (MDGs) of reducing child mortality by two-thirds. Specifically, from 1999 to 2022, neonatal and under-five mortality rates decreased from 47 deaths to 20 deaths per 1,000 live births and from 147 to 49 deaths per 1,000 live births, respectively [8, 9].

To improve the access to basic health services, Tanzania implemented a primary health service development program (2007–2017) by renovating and building at least one dispensary per village and one health center per municipal unit throughout the country [10].

Despite the presence of exemption and waiver policies witch protect poor and vulnerable groups (e.g. pregnant women, children and the elderly), the implementation of these policies remains ineffective in many areas of Tanzania [11, 12].

Tanzania is divided in 21 regions, housing 369 hospitals [13]. The Iringa District Council (Iringa DC) is a district in the Iringa Region in southern Tanzania, where The Tosamaganga Voluntary Agency Hospital (Tosamaganga Hospital) operates since ‘80s.

In Iringa region, like other Tanzanian regions, the hospital emergency departments often remain the only or the main points of access to health-care services. The hospitalization rates after an access to Emergency department may reflect the lack of preventive and primary care for conditions that could be managed and treated in a setting other than a hospital, in particular for pediatric patients [14].

Therefore, it may be important to assess for which conditions a pediatric population in a developing country uses hospital’s emergency services and may need hospitalization, to support health policy choices to promote interventions for the purpose of improving outcomes for children with acute and critical illnesses.

This study aims to describe the clinical profile and outcomes of pediatric presentations to Tosamaganga Hospital OPD departments over a 9-month period, and to identify predictors of hospitalization in a pediatric OPD care services in a LMIC.

## Methods

### Study design and population

We retrospectively collected data on children population (aged 0–13 years old) accessing to the OPD (pediatric and general) at Tosamaganga Hospital, over a 9-month period, within the period 1 January − 30 September 2022. The upper age limit for the inclusion of patients was 13 years old, because hospital policy dictates that from the age of 14 years old, patients have been seen at general Outpatient Department (OPD) and admitted to the adult wards. Data were collected for all patients who accessed the OPD services, both general (with non-pediatric trained staff) and pediatric (with pediatric trained staff).

We have obtained from the institution of Tosamaganga Hospital the necessary permissions to carry out the study. The Health Management Team of Tosamaganga Voluntary Agency Hospital formally approved the ethical aspects of the study.

### Setting

The Tosamaganga Voluntary Agency Hospital (Tosamaganga Hospital) owned by the Diocese of Iringa is located about 16 km from the capital city, Iringa. The Region spans an area of 35,503 km2 and it is divided into 5 districts, namely Iringa Urban, Iringa DC, Kilolo, Mufindi District Council and Mafinga. The population of the region exceeds 1,000,000.

The hospital has a capacity of 192 beds and in 2022 it registered about 33,000 outpatient visits, 9,500 admissions and 3,500 deliveries. In addition to inpatient activities, the hospital provides outpatient services (known as Outpatient Department, OPD), which operates 24/7 and caters to patients from outside the hospital. An OPD dedicated exclusively to pediatric patients (pediatric OPD) is present since 2019, where patients are examined by a clinical officer trained in the management of pediatric acute conditions. The pediatric OPD operates from 8 a.m. to 4 p.m. approximately, Monday to Friday. Outside these hours (nights and holidays), patients are examined in the general OPD.

### Data collected

The data examined in this study were extrapolated from the Health Information Management System (HMIS) currently in use at Tosamaganga Hospital. The following data were collected from the HMIS: gender, age, area of residence, whether insured or uninsured, visiting clinic, access symptoms, outcome (discharge, admission, transfer, or death).

The variable of the area of residence was classified based on the distance (in kilometers) to travel from the village of residence to the hospital. For statistical analysis, the accesses were divided according to the districts of greatest influx: Iringa DC, Iringa Urban, and the other areas.

Information on access symptomatology was classified based on the diagnosis reported by the health professional who visited the child, dividing them into two main medical categories:

1. Non-traumatic disorders: infectious diseases such as gastrointestinal infections (viral, bacterial or parasitic), lower respiratory tract diseases (pneumonia, bronchitis, bronchiolitis), skin infections, sensory organ-related infections (tonsillitis, sinusitis, otitis and conjunctivitis), urinary tract infections, malaria, sepsis, meningitis and encephalitis. Non-infectious diseases include malnutrition, sickle cell anaemia, epilepsy, febrile seizures, heart failure due to congenital heart disease or rheumatic disease, umbilical hernia, appendicitis, and neonatal jaundice.
2. Injury-related presentations, classified as an accident, trauma, injury, violence, ingestion and inhalation of foreign bodies, or burns.

Additionally, information about transfer to another facility and death was also collected.

The primary outcome studied was hospital admission to Tosamaganga Children Ward.

### Statistical analysis

All the analyses are referred to the accesses to the OPD service as statistical analysis unit.

In particular, the descriptive analysis presents absolute and relative frequencies for categorical variables, while the mean, standard deviation, median, interquartile range of the distribution are shown for continuous variables.

Differences in the proportions of the distribution of the qualitative variables, in the mean and median values of the distribution of the quantitative variables, between OPD services accesses that did or did not result in a hospital admission, death, or transfer, were assessed.

The chi-square test was used for qualitative variables, while for quantitative variables the non-parametric Wilcoxon-Mann-Whitney test was performed, as the assumption of normality distribution was not formally verified.

The *p*-values presented in the analyses are derived from two-tailed tests (95% confidence interval).

The association between the outcome (hospitalization) and potential confounding covariates (demographic, socio-contextual and clinical factors) was evaluated using univariate and multivariate logistic regression models.

As each subject can potentially experience multiple access to OPD service, logistic regression models with robust standard errors were performed. In this way, we took into account that the individual observations were not independent from each other, allowing for intragroup correlation and accounting for heteroscedasticity.

Multivariate model was carried out including age in months and sex as a priori risk factors, and all the variables that at univariate analysis were associated with the outcome with a *p*-value < 0.10.

Statistical data analyses were performed using Stata 16 software [15].

## Results

Among the 9-month study period, a total of 2,810 children were visited, accounting for 4,324 accesses in the OPD. Of these, 2,331 (53.9%) were males and 1,993 were females (46.1%), with a male-to-female ratio of 1.17 to 1. The mean age at access was 43.2 months (SD 41.6), with a median age of 27.2 months (IQR 9.6–68.6).

Among children at visit, 185 (4.3%) were in neonatal age (0–28 days), 1,094 (25.3%) in post-neonatal age (29–365 days), 1,831 (42.4%) in pre-scholar age (1–4 years) and 1,214 (28.1%) in scholar age (6–13 years).

Most of accesses were referred to children who came from the districts of Iringa DC (68.1%) and Iringa Urban (25.4%), 6.5% from other districts.

The mean distance to the hospital was 33.1 km (SD 39.7), with a median of 20 km (IQR 12–36). The average travel time to the hospital was 43.4 min (SD 42.7), with a median of 31 min (IQR 19–53).

Out of the total number of accesses, 70.8% of patients did not have health insurance, whereas 29.2% had health insurance policies covering the expenses of services provided.

Overall, 87.9% of accesses to OPD were performed from Monday to Friday and 12.1% on weekend. Pediatric-trained personnel attended to 61.8% of the accesses, while the remaining 38.2% were attended to by personnel without pediatric training.

Data on the presenting symptoms of the access were available in 93.0% of the cases, while for 7.0% of accesses, there was no information about the admission symptomatology.

Medical symptomatology accounted for the reason for presentation in 90.5% of cases, while 9.5% were due to injury. The most common reasons for presentation were sensory organ-related disorders (symptomatology involving nose, ear, eye, and throat for 19.2%), followed by genitourinary symptoms (14.1%), post-therapy follow-up, post-hospitalization or anthropometric and neurodevelopmental assessment (12.3%), and gastrointestinal disorders (10.9%).

For inpatients, on the other hand, the most common admission diagnoses were related to malnutrition (20.1%), respiratory disorders (12.2%), osteo-articular injuries (11.2%) and skin disorders and infections (9.0%).

Among all the OPD accesses,, 503 (11.6%) were admitted at Tosamaganga Hospital, 17 (0.4%) were transferred to other facilities to allow further investigation and treatment, and 17 (0.4%) died during hospitalization. Among the 503 hospitalized patients, 14 (2,8%) were referred to higher centers or developed a need for intensive care. The causes of admission to OPD for the 17 deaths, were the following: 8 (47.1%) respiratory disease, 4 (23.5%) malnutrition, 2 (11.8%) genitourinary infection, 1 (5.9%) heart failure, 1 (5.9%) skin infection, 1 (5.9%) injury.

**Fig. 1:**
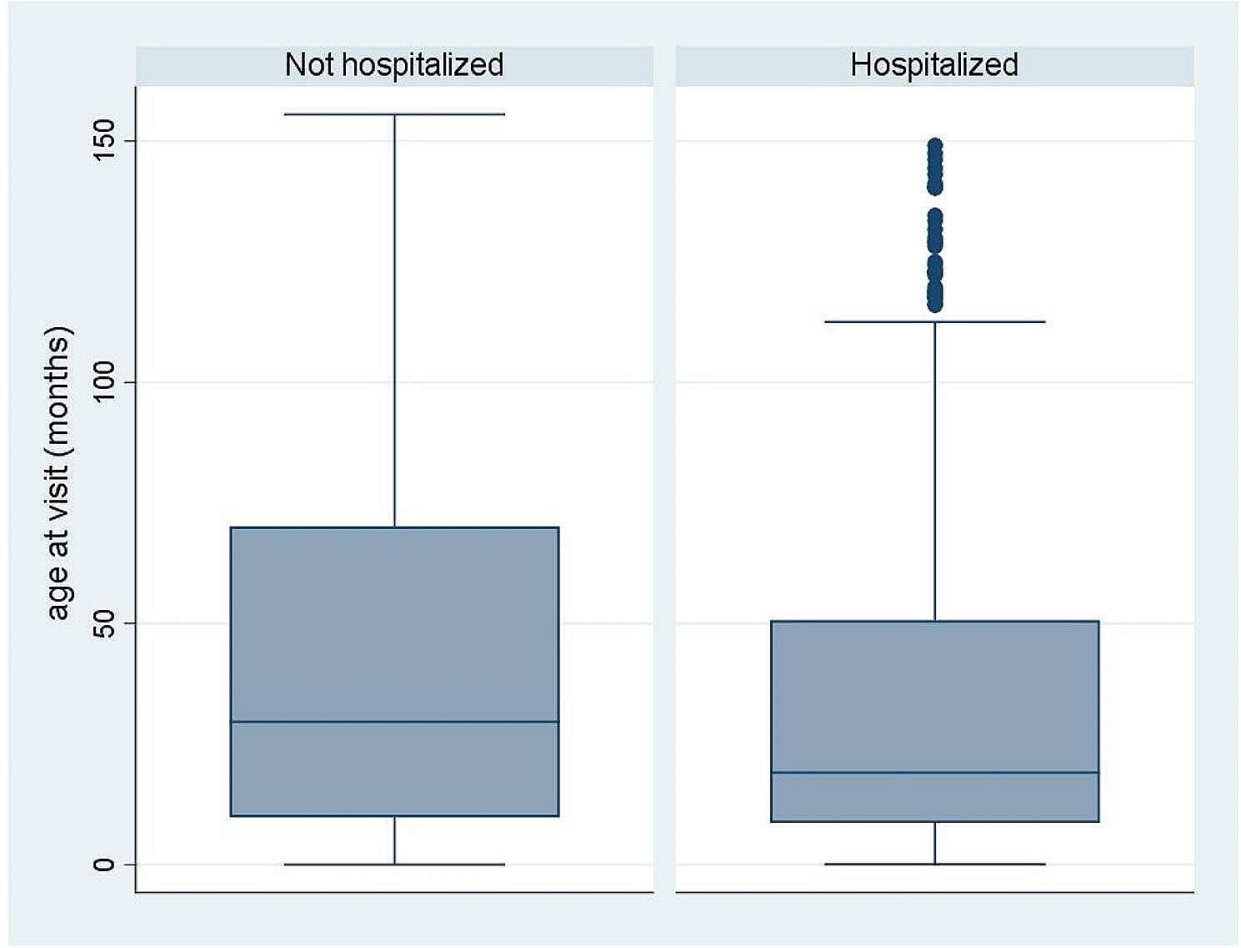
Age at visit of subjects admitted to OPD, by hospitalization

The children admitted to the hospital were in average younger (*p* < 0.001) than those not admitted: 44.5 (SD 42.1) vs. 34.7 (SD 36.8) months old (median 30 [IQR 10–70] vs. 19 [IQR 9–51]) (Fig. 1).

Table 1 summarizes the demographic, social, and clinical characteristics of subjects admitted to OPD by hospitalization.

**Table 1:** Demographic, social, and clinical characteristics of subjects admitted to OPD, by hospitalization

		Hospitalization						
		Not		Yes		Total		
		N.	%	N.	%	N.	%	*P*-value
Sex	Males	2,041	87.6	290	12.4	2,331	100	0.07
	Females	1,780	89.3	213	10.7	1,993	100	
Insurance	Not	2,605	85.1	455	14.9	3,060	100	< 0.001
	Yes	1,216	96.2	48	3.8	1,264	100	
District of residence	Iringa urban	1,015	92.4	84	7.6	1,099	100	< 0.001
	Iringa DC	2,591	88.0	353	12.0	2,944	100	
	Other	215	76.5	66	23.5	281	100	
Presence of paediatric trained staff	Not	1,319	79.8	334	20.2	1,653	100	< 0.001
	Yes	2,502	93.7	169	6.3	2,671	100	
Burn / Wound	Not	3,813	88.8	482	11.2	4,295	100	< 0.001
	Yes	8	27.6	21	72.4	29	100	
Cardiovascular	Not	3,806	88.5	494	11.5	4,300	100	< 0.001
	Yes	15	62.5	9	37.5	24	100	
Constitutional / malnutrition	Not	3,757	90.7	384	9.3	4,141	100	< 0.001
	Yes	64	35.0	119	65.0	183	100	
Fever	Not	3,644	89.0	452	11.0	4,096	100	< 0.001
	Yes	177	77.6	51	22.4	228	100	
Gastrointestinal	Not	3,437	89.2	416	10.8	3,853	100	< 0.001
	Yes	384	81.5	87	18.5	471	100	
Respiratory	Not	3,647	89.5	427	10.5	4,074	100	< 0.001
	Yes	174	69.6	76	30.4	250	100	
Dermatological	Not	3,471	87.7	488	12.3	3,959	100	< 0.001
	Yes	350	95.9	15	4.1	365	100	
Genitourinary	Not	3,228	86.9	485	13.1	3,713	100	< 0.001
	Yes	593	97.0	18	3.0	611	100	
Neurological	Not	3725	88.4	488	11.6	4,213	100	0.53
	Yes	96	86.5	15	13.5	111	100	
Eye, ear, nose, throat medical complaints	Not	3,008	86.1	485	13.9	3,493	100	< 0.001
	Yes	813	97.8	18	2.2	831	100	
Ingestion / Inhalation	Not	3,814	88.4	498	11.6	4,312	100	0.001
	Yes	7	58.3	5	41.7	12	100	
Injury	Not	3,593	88.9	448	11.1	4,041	100	< 0.001
	Yes	228	80.6	55	19.4	283	100	
Wound / Violence	Not	3,813	88.3	503	11.7	4,316	100	0.30
	Yes	8	100.0	0	0.00	8	100	
Month of the admission to OPD	January	251	86.9	38	13.1	289	100	< 0.001
	February	337	91.3	32	8.7	369	100	
	March	507	85.9	83	14.1	590	100	
	April	499	83.9	96	16.1	595	100	
	May	461	88.7	59	11.3	520	100	
	June	417	89.5	49	10.5	466	100	
	July	395	92.3	33	7.7	428	100	
	August	488	90.9	49	9.1	537	100	
	September	463	87.9	64	12.1	527	100	

A higher percentage of hospitalization was observed among males than among females (12.4% vs. 10.7%, *p* = 0.07).

A higher percentage of hospitalizations was observed among those who did not have insurance than among those who did (14.9% vs. 3.8%, *p* < 0.001).

A higher percentage of inpatients was observed among those who came from the districts furthest from the hospital, compared to those who came from the rural Iringa district and the urban Iringa district, 23.5%, 12.0%, 7.6% (*p* < 0.001), respectively.

**Fig. 2:**
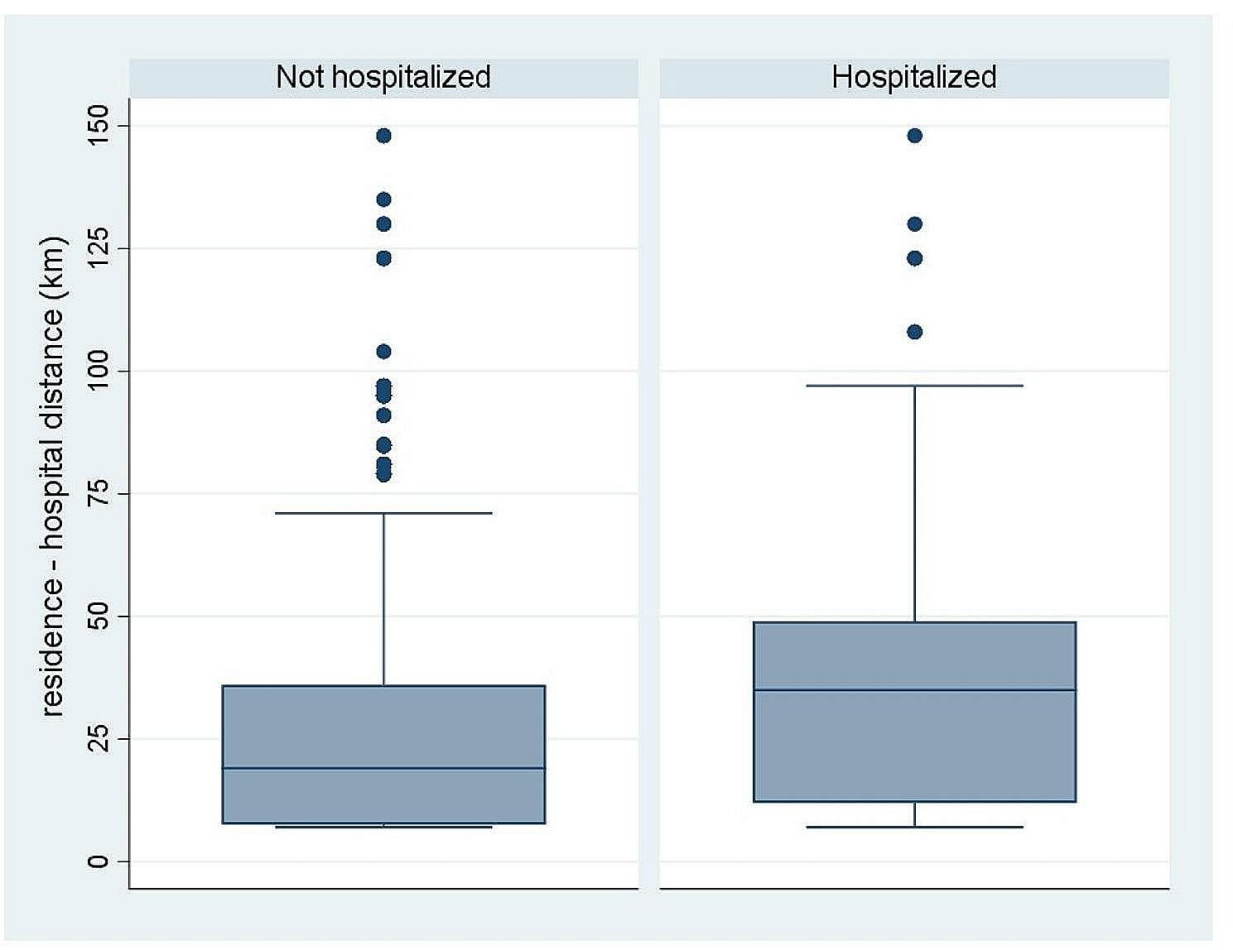
Distance from residence to hospital of subjects admitted to OPD, by hospitalization

An association between the distance of residence from the hospital and hospitalization was confirmed (Fig. 2) by the finding that the distance in kilometers was higher (*p* < 0.001) among hospitalized than not hospitalized (mean 46.9 [SD 55.2] vs. 31.3 [36.9] km (median 36 [IQR 12-61.5] vs. 19 [IQR 12–36)).

A lower percentage of inpatients was observed among those who were visited in the outpatient clinic in the presence of pediatric-trained health personnel, compared to those who were visited by non-pediatric-trained personnel (6.3% vs. 20.2%, *p* < 0.001).

In relation to symptoms at the time of admission to OPD, a higher percentage (*p* < 0.001) of inpatients was observed among those who had or did not have one of the following symptoms: burn/wound (72.4% vs. 11.2%), cardiovascular (37.5% vs. 11.5), constitutional/malnutrition (65.0% vs. 9.3%), fever (22.4% vs. 11.0%), gastrointestinal (18.5% vs. 10.8%), respiratory (30.4% vs. 10.5%), ingestion/inhalation (41.7% vs. 11.6), injury (19.4% vs. 11.1%). On the contrary, we observed a lower percentage (*p* < 0.001) of inpatients if the following symptoms at access to OPD were present and not absent: dermatological (4.1% vs. 12.3%), genitourinary (3.0% vs. 13.1%), eye, ear, nose, throat medical complaints (2.2% vs. 13.9%).

Table 2 summarizes the results of univariate logistic regression models performed to evaluate the association between demographic, social, and clinical characteristics of subjects admitted to OPD with the risk of hospitalization. A higher risk of hospitalization for the admissions to OPD was observed for the subjects without insurance (OR 4.42), coming from more distant districts (OR 3.71) and from Iringa rural (OR 1.65) compared to Iringa urban, coming from a village with more distance from residence to hospital (for each additional km traveled OR 1.01), for those visited by a not pediatric trained staff (OR 3.75) and who accessed to OPD for the following symptoms: burn/wound (OR 20.77), cardiovascular (OR 4.62), constitutional/malnutrition (OR 18.19), fever (OR 2.32), gastrointestinal (OR 1.87), respiratory (OR 3.73), ingestion/inhalation (OR 5.47), injury (OR 1.93). Furthermore, males had higher risk (*p* = 0.07) compared to women, while a lower risk of hospitalization was found for age (in months) increase (OR 0.994), for those with genitourinary symptoms (OR 0.20) and for children who had access to OPD in July (OR 0.55, *p* = 0.02), February (OR 0.63, *p* = 0.06), and August (OR 0.66, *p* = 0.08) compared to January, which was considered the reference month.

**Table 2:** Factors associated to hospitalization. Results of univariate logistic regression models

VARIABLES		Odds Ratio	95CI%	*P*
age at visit	each 1 month more	0.994	0.991–0.996	< 0.001
sex	females	1.00	-	-
	males	1.19	0.97–1.45	0.094
insurance	yes	1.00	-	-
	not	4.42	3.20–6.12	< 0.001
district of residence	Iringa urban	1.00	-	-
	Iringa DC	1.65	1.28–2.12	< 0.001
	other districts	3.71	2.57–5.36	< 0.001
distance from residence to hospital	each 1 km more	1.007	1.005–1.009	< 0.001
presence of paediatric trained staff	yes	1.00	-	-
	not	3.75	3.07–4.58	< 0.001
burn / wound	not	1.00	-	-
	yes	20.77	7.64–56.45	< 0.001
cardiovascular	not	1.00	-	-
	yes	4.62	1.96–10.93	< 0.001
constitutional / malnutrition	not	1.00	-	-
	yes	18.19	13.14–25.18	< 0.001
fever	not	1.00	-	-
	yes	2.32	1.67–3.23	< 0.001
gastrointestinal	not	1.00	-	-
	yes	1.87	1.44–2.43	< 0.001
respiratory	not	1.00	-	-
	yes	3.73	2.78–5.01	< 0.001
ingestion / inhalation	not	1.00	-	-
	yes	5.47	1.73–17.31	0.004
injury	not	1.00	-	-
	yes	1.93	1.41–2.66	< 0.001
month of the admission to OPD	January	1.00	-	-
	February	0.63	0.38–1.02	0.062
	March	1.08	0.71–1.64	0.713
	April	1.27	0.84–1.93	0.262
	May	0.85	0.54–1.32	0.457
	June	0.78	0.49–1.24	0.285
	July	0.55	0.34–0.91	0.019
	August	0.66	0.42–1.05	0.079
	September	0.91	0.59–1.42	0.687

**Fig. 3:**
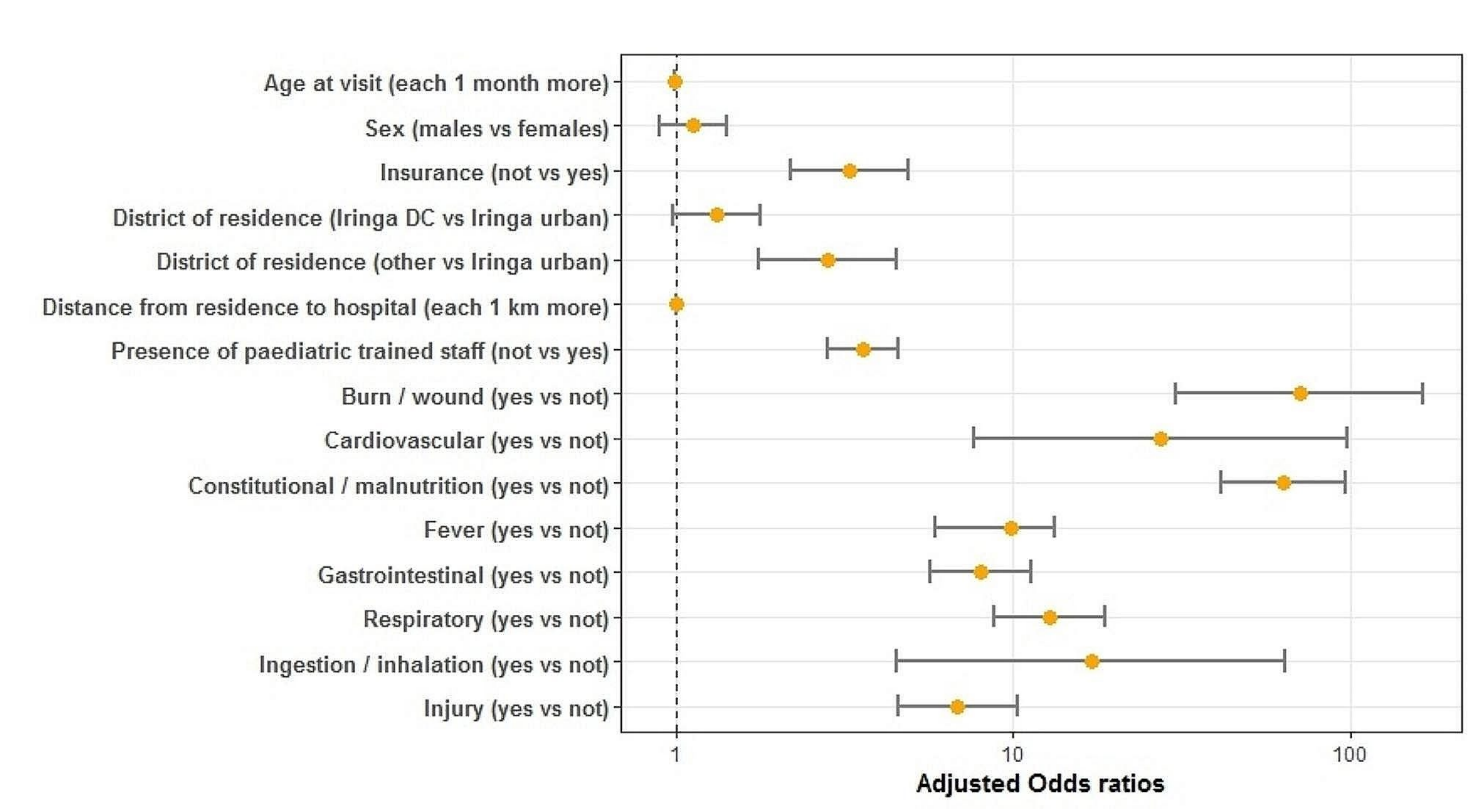
Demographic, socio-contextual and clinical factors associated with hospitalization. Multivariate logistic regression model

Multivariate logistic regression analysis (Fig. 3) confirm the results of univariate logistic regression analysis, showing a higher (*p* < 0.001) risk of hospitalization for the admissions to OPD without insurance (OR 3.26, 95%CI 2.18–4.89), coming from more distant districts (OR 2.83, 95%CI 1.76–4.53), for distance (in kilometers) from residence to hospital increase (OR 1.003, 95%CI 1.001–1.006), for those visited by a not pediatric trained staff (OR 3.58, 95%CI 2.82–4.55), and who accessed to OPD for the following symptoms: burn/wound (OR 70.63, 95%CI 30.41-164.06), cardiovascular (OR 27.36 95%CI 7.68–97.44), constitutional/malnutrition (OR 62.71, 95%CI 41.04–95.82), fever (OR 9.79, 95%CI 5.86–13.18), gastrointestinal (OR 8.01, 95%CI 5.69–11.28), respiratory (OR 12.86, 95%CI 8.80-18.79), ingestion/inhalation (OR 17.00, 95% CI 4.53–63.77), injury (OR 6.84, 95%CI 4.54–10.32). A lower risk of hospitalization was found for age (in months) increase (OR 0.993, 95%CI 0.989–0.996).

## Discussion

In this study, we present the first dataset on pediatric emergency visits to an OPD of a district-level hospital in rural Tanzania.

Overall, we observed a hospitalization rate of approximately 12% among the OPD, which was lower than the rates previously reported in other studies [16–19]. Instead, the mortality rate (0.4%) was higher than reported in high-income countries (1.5/100,000 visits) [20], but it was comparable to the rate found by Enyuma et al. [16], where in their study (0.5%), and even lower compared to other sub-Saharan countries like Nigeria where the rates was ranged 2-17.5% [21–25] or Cameroon where Chiabi et al. found a mortality rate of 1,6 − 1,9% [19] and Chelo et al. found a rate of 5,76% [26]. Our findings revealed a higher risk of hospitalization for the admissions to OPD among patients without insurance, those from more distant districts, those attended by a non-paediatric trained staff and those accessing the OPD for burn/wound, cardiovascular, constitutional/malnutrition, fever, gastrointestinal, respiratory, ingestion/inhalation, injury. Conversely, we observed a lower risk of hospitalization when the age of children increased.

The findings suggest that living in suburban or otherwise remote areas, far from health centers, is a risk factor for hospitalization. Juran et al. found that 92.5% of the sub-Saharan African population lived within 2 h of a major hospital for surgical procedures [27]. The increased risk is likely due to the greater distance to the hospital, which may lead to a delayed access to OPD for the children. Manongi and colleagues in Tanzania found that hospitalised children who came from areas less than 3 h away from the hospital had a mortality rate of 3.4% compared to 8.0% for children who came from areas more than 3 h away [28]. These delays can be attributed to socioeconomic factors related to high costs of transportation, inadequate transportation infrastructures and vehicles, which in turn may result in the exacerbation of the disease not treated in a timely manner. This observation may also reflect the large number of challenges in traveling to the hospital that children face even when they are seriously ill [29]. A WHO report on quality of care recommends timely referral for every child with conditions that cannot be managed effectively at first-level facilities [30].

Previous studies highlighted that the reasons for late presentations at pediatric emergency units includes poor identification of early sign of disease severity by care givers of low level health facilities, high costs of hospital treatments as patient pay out of pocket, poor health seeking behaviors and beliefs in remedies with unproven efficacy [31, 32].

Although challenging and costly, the establishment of an efficient emergency call and ambulance transport service would be crucial to ensure that critically ill children from both suburban and urban areas have timely access to OPD. Safe transport is required by many sick children seen in primary care facilities to referral hospitals. However, implementing such a service may not always be feasible, and this need to be balanced against the potential risk of transport, distance to referral hospital, costs and the needs of other patients. In Tanzania, transportation from primary care health posts to hospitals is even more difficult, and other modes of emergency transport are employed; these included bicycles with trailers, tricycles with platforms, motorboats and ox carts [33].

Another relevant observation of the present study is that owning health insurance is a protective factor for hospitalization risk. The existing health insurance schemes in Tanzania only cover medical costs at healthcare facilities, but do not compensate patients for travel and time costs incurred in accessing care, resulting in financial burden on households and delayed access to care [34]. The health financing system in Tanzania is highly fragmented involving different resource providers including general taxation (34%), private donors, non-governmental organizations (NGOs), foreign states etc. (36%), direct payments (22%) and health insurance contributions (8%) [35]. According to a 2018 analysis of the health sector only 33% of Tanzanians are covered by health insurance, leaving the remaining two-thirds 2/3 of population exposed to financial arising from direct health care payments [35]. This finding could be partly correlated with the above, assuming that the family with health insurance is in better economic condition and thus can more easily afford transportation to the hospital. In addition, by not having to pay for the health service, the parent would tend to bring the child in early for a medical examination as shown by Huang at al. in Taiwan [36], with opportunities then for health personnel to intervene early on the ongoing condition. Moreover, we can hypothesize that the higher level of education of families with health insurance may also play a role, being more aware of the warning signs/symptoms of the child’s pathology. As demonstrated by Agelebe at al [37]., children who were socially disadvantaged presented significantly later to the hospital than their non-socially disadvantaged counterparts following onset of illnesses. Low utilization of healthcare services due to delay in making decision and delay in assessing medical services is because of the ripple effects of unemployment and poverty [37].

Our study showed that children were more likely to be hospitalized if they had cardiovascular, constitutional, neurological, gastrointestinal, respiratory symptoms, or had burns. In the literature other studies in LMICs report found similar rates. Specifically, in LMICs the prevalence of preventable communicable diseases (such as malaria, pneumonia and diarrheal diseases) and acute and chronic malnutrition is high in sub-Saharan African Countries [19, 21, 26, 38–41].

In addition to the high burden of pediatric infectious disease, there is at present an increasing incidence of non-communicable and hereditary diseases and their complications, that require special care by specialized personnel. Such symptomatology in the pediatric patient takes on different peculiarities and complexity than in the adult one, thus emphasizing that specific expertise is needed [16, 23, 42–44].

Our study supports this hypothesis, as it indicates that visiting an outpatient clinic not specifically dedicated to pediatric care is predictive of an increased risk of hospitalization. Indeed, our findings may suggest that better management of the pediatric patient even at the OPD level could reduce avoidable hospitalizations, which can cause stress and economic burden to families, as well as increase the risk of hospital-acquired infections. Several studies have shown that children that arrived at emergency departments during off hours tend to experience longer stays [21, 45–46]. This is attributed to the unavailability of highly skilled personnel and certain investigations during such periods of the day [31, 32]. Also, healthcare personnel trained in pediatric emergency medicine principles are shown to reduce childhood mortality in LMICs likely through dissemination of education, practice patterns, and advocacy measures [47].

Training health personnel in early identification of critical illness is a crucial step in improving disease prognosis, as shown by several studies [48, 49]. Priority should be given to training in the early recognition and management of pediatric conditions that most commonly lead to death in the local area. This issue holds particular significance in LMICs [50], such as Tanzania, where, like many sub-Saharan Africa countries, there is a shortage of trained healthcare professionals with an estimated 3 doctors and 39 nurses per 100 000 inhabitants in the country [51, 52].

Early triage assessment and prompt identification of signs of critical illness, and rapid initiation of appropriate treatment should be top priorities for all hospitals providing emergency care for children [53]. However, according to the study of Ardsby et al. [54], only 9.1% of the hospitals reported specific triage protocols for children < 5 years of age. Inadequate triage is a widespread problem in LMICs and represents an important challenge in addressing emergency conditions in hospitals [55, 56].

Finally, our study revealed that the children admitted to hospital were in average younger than not admitted (about 10 months older), a result in agreement with what other studies have observed [18]. However, in our study are included children who came for follow up for post-therapy, post hospitalization or anthropometric and neurodevelopmental assessment (12.3% of the total population examined).

### Strengths

This study provides information about pediatric emergency visits to an OPD of a district-level hospital in Tanzania on a large sample of children, a still under-researched field of research that needs as much work as possible for reduce preventable deaths.

Moreover, the availability of data collected through dedicated software is another strength of our study. Optimization of data recording is an important area on which to focus resources to obtain increasingly accurate data. The data collection was done through the new HMIS introduced in early 2022 at Tosamaganga Hospital. The availability of this software allowed to collect good quality information in terms of accuracy, reliability, consistency. In fact, the current data recording system that is used in the hospital allows for good data recording with minimal gaps that are likely to be reduced over time. The HMIS proved to be effective and easily usable in the data collection process, confirming that having better data quality simplifies the monitoring of health care delivery and evaluation of the impact of health interventions [57].

### Limitations

This is a single-center study of a district-level hospital; it may not represent the rest of Tanzania or other LMICs, but it is reasonable to assume that our data provide some useful information about pediatric emergency care at the local and national levels that can help optimize the distribution and use of resources, as well as plan more appropriate feasible and effective interventions to improve pediatric emergency care within an integrated system of care.

For some variables we could not collect information for all subjects because they had not been registered in the electronic database, which became operational just in January 2022, when the study started. Furthermore, we could not collect information on the last three months of 2022, a limitation that did not allow to assess whether there are seasonal differences on the outcome. However, we performed analyses by month of admission to OPD.

Moreover, data on comorbidities different from those collected are not reported in the OPD database, thus in the inpatient database they are mentioned inconsistently and, therefore, could not be analyzed.

This would be extremely valuable information to include in multivariate analysis, as underlying conditions such as malnutrition, HIV, and other infectious or chronic diseases have been shown to be associated with need for hospitalization and mortality [58, 59]. Systematic collection of major comorbidities should be pursued to be able to properly interpret the data to improve care and optimize organization and resource utilization.

Finally, it was not possible to define the aetiological diagnosis of the diseases due to instrumental and laboratory diagnostic limitations.

## Conclusions

The findings of higher risk of hospitalization for children without health insurance, and living far from the hospital underline the necessity to promote health education and policies aimed at extending the universality of health care, through the implementation of primary care as close as possible to the population, in particular in little villages. The establishment of an efficient emergency call and transport system could support this healthcare priority.

The observation that children visited by staff including health care personnel specifically trained on pediatric patient management confirm the necessity to improve the quality of care provided to children with acute and critical illness, avoiding admissions for pathologies that could be treated in other health settings, such as primary care, if timely evaluated. The presence of a triage system, not currently present at Tosamaganga Hospital, could facilitate a more specific access to emergency care.

The experience of the Tosamaganga Hospital, with the presence of an electronic database suggests that optimizing data recording is central in the development of a health care system, because a monitoring system can produce information useful for health care providers and for policy makers. In fact, the possibility of identifying some relevant risk factors of hospitalization, as our study did, may support the health policy choices useful to promote interventions such as family health education, training of health staff, implementation of a triage system and of an emergency care transport system useful to the development of pediatric care system in Tanzania and in other medium-low-resource country, improving outcomes for children with acute and critical illnesses.

### Electronic supplementary material

Below is the link to the electronic supplementary material.

Supplementary Material 1

## Data Availability

The database that support the findings of this study were used under license by Tosamanga Voluntary Agency Hospital for the current study, and are thus not publicly available. Specifical statistical analysis can be requested and agreed with Tosamanga Voluntary Agency Hospital. However, all data relevant to the study have been included in the paper.

## Abbreviations

CI: Confidence Interval
DC: District Council
HIV: Human immunodeficiency virus
HMIS: Health Information Management System
IQR: Interquartile Range
KM: Kilometer
LMIC: Low and Middle-Income Country
MDG: Millennium Development Goal
N: Number
NGO: Non-Governmental Organization
OPD: Outpatient Department
OR: Odds Ratio
P: *P*-Value
SD: Standard Deviation
VS: Versus
WHO: World Health Organization
