# Supplementary material for: Factors associated with hospitalization in a pediatric population of rural Tanzania: findings from a retrospective cohort study
Source: Ital J Pediatr. 2024 Mar 18;50:53. doi: 10.1186/s13052-024-01622-z (PMC10949679; doi:10.1186/s13052-024-01622-z)
Supplement: Supplementary file 1 — Supplementary Material 1 [file 13052_2024_1622_MOESM1_ESM.docx]

Professor Giovanni Corsello

Editor-in-Chief

Italian Journal of Pediatrics

14 December 2023

Dear Professor Giovanni Corsello,

Please find enclosed the manuscript entitled “Factors associated with hospitalization in a pediatric population of rural Tanzania: findings from a retrospective cohort study”, by Vincenzo Mancini, Martina Borellini, Paolo Belardi, Maria Carolina Colucci, Emanuel Yuda Kadinde, Christina Mwibuka, Donald Maziku, Pasquale Parisi, and Anteo Di Napoli, to be considered for publication as an original research article in Italian Journal of Pediatrics.

The present retrospective cohort study, based on 4,324 accesses at Tosamaganga Hospital (Tanzania) of 2,810 children aged 0-13, aims to describe the characteristics of paediatric presentations to an Out Patient Department and to identify the predictive factors of hospitalization.

We found a higher risk of hospitalization for children without health insurance, living far from the district capital, and for those did not visit by staff including health care personnel specifically trained on pediatric patient management.

We think that our study would be of interest to your readers because the possibility of identifying some relevant risk factors of hospitalization, as our study did, may support choices of health policies useful to the development of pediatric care system in Tanzania and in other medium-low-resource country, improving outcomes for children with acute and critical illnesses.

The manuscript is not under consideration for publication in any other journal.

All listed authors do not have any financial or other relationships that might lead to a conflict of interest, and have approved the manuscript before submission, including the names and order of authors.

The study has been formally approved for ethical aspects by the Health Management Team of Tosamaganga Voluntary Agency Hospital.

In consideration of their expertise, we would like to suggest the following researchers as possible reviewers for the article:

- Carlo Di Lorenzo, Children's Hospital in Columbus, Columbus, USA
- Raffaele Falsaperla, Policlinico -Vittorio Emanuele Hospital, Catania, Italy
- Ettore Piro, University Palermo, Palermo, Italy
- Martino Ruggieri, Catania University, Catania , Italy
- Pasquale Striano, Genova University, Genova, Italy
- Caterina Strisciuglio, Luigi Vanvitelli Campania University, Naples, Italy
- Alberto Verrotti, Perugia University, Perugia, Italy

Thank you in advance for your consideration.

Best regards

Vincenzo Mancini, Martina Borellini, Paolo Belardi, Maria Carolina Colucci, Emanuel Yuda Kadinde, Christina Mwibuka, Donald Maziku, Pasquale Parisi and Anteo Di Napoli
